# A new pseudopolymorph of perchlorinated neo­penta­silane: the benzene monosolvate Si(SiCl_3_)_4_·C_6_H_6_

**DOI:** 10.1107/S2056989020000900

**Published:** 2020-01-31

**Authors:** Jan Tillmann, Hans-Wolfram Lerner, Michael Bolte

**Affiliations:** a Institut für Anorganische und Analytische Chemie, Goethe-Universität Frankfurt, Max-von-Laue-Strasse 7, 60438 Frankfurt am Main, Germany

**Keywords:** crystal structure, co-crystal, polymorphism, solvate

## Abstract

A new pseudopolymorph of dodeca­chloro­penta­silane, namely a benzene monosolvate, Si_5_Cl_12_·C_6_H_6_, is described. There are two half mol­ecules of each kind in the asymmetric unit. Both Si_5_Cl_12_ mol­ecules are completed by crystallographic twofold symmetry. One of the benzene mol­ecules is located on a twofold rotation axis with two C—H groups located on this rotation axis. The second benzene mol­ecule has all atoms on a general position: it is disordered over two equally occupied orientations. No directional inter­actions beyond normal van der Waals contacts occur in the crystal.

## Abstract 2

A new pseudopolymorph of dodeca­chloro­penta­silane, namely a benzene monosolvate, Si_5_Cl_12_^.^C_6_H_6_, is described. There are two half mol­ecules of each kind in the asymmetric unit. Both Si_5_Cl_12_ mol­ecules are completed by crystallographic twofold symmetry. One of the benzene mol­ecules is located on a twofold rotation axis with two C—H groups located on this rotation axis. The second benzene mol­ecule has all atoms on a general position: it is disordered over two equally occupied orientations. No directions inter­actions beyond normal van der Waals’ contacts occur in the crystal.

## Chemical context   

**Figure 1:**
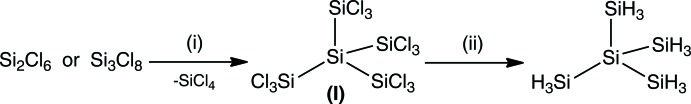
Amine-induced disproportionation of Si_2_Cl_6_ and Si_3_Cl_8_: (i) + NMe_3_, or NMe_2_Et, or NEt_3_ in benzene at room temperature; (ii) + LiAlH_4_ in diethyl ether at room temperature

Since the 1980s, silicon hydrides, such as Si(SiH_3_)_4_, have attracted considerable attention as precursors for the liquid phase deposition (LPD) of silicon thin films (Nishimura *et al.*, 1985 ▸). In this context it should be noted that the perchlorinated neo­penta­silane Si(SiCl_3_)_4_ (Si_5_Cl_12_) is easily accessible in large amounts by the amine-induced disproportionation (Meyer-Wegner *et al.*, 2011 ▸; Tillmann *et al.*, 2012 ▸) of perchloro­polysilanes, *e.g.* Si_2_Cl_6_ or Si_3_Cl_8_ (Meyer-Wegner *et al.*, 2011 ▸; Urry, 1970 ▸). Subsequent hydrogenation of Si(SiCl_3_)_4_ (I) then yields the neo­penta­silane Si(SiH_3_)_4_, which can be used as an LPD agent (Cannady & Zhou, 2008 ▸) (see Fig. 1 ▸).
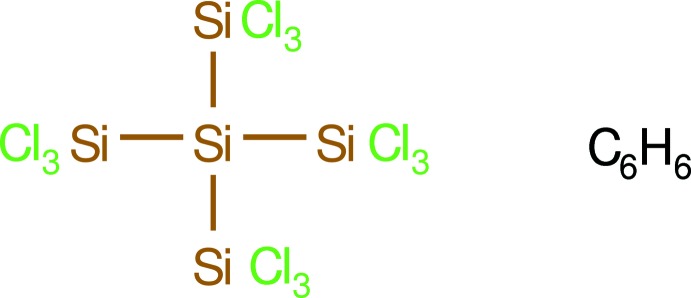


In this paper we describe the structure of a new pseudo-polymorph of perchlorinated neo­penta­silane (I), namely the benzene monosolvate Si(SiCl_3_)_4_·C_6_H_6_, and make a comparison of its structure with those of Si(SiCl_3_)_4_ (Meyer-Wegner *et al.*, 2011 ▸) and Si(SiCl_3_)_4_·SiCl_4_ (Fleming, 1972 ▸).

## Structural commentary   

**Figure 2:**
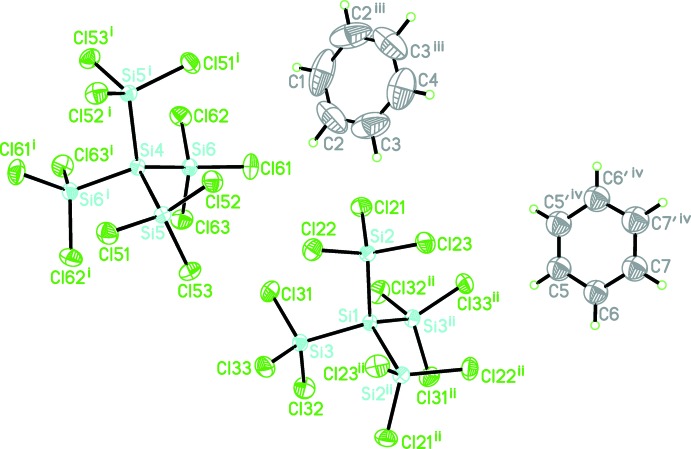
A perspective view of the title compound. Displacement ellipsoids are drawn at the 50% probability level. Symmetry codes: (i) −*y*, −*x*, −*z* + 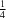; (ii) 1 − *y*, 1 − *x*, −*z* + 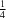; (iii) −*x*, *y*, −*z*; (iv) 1 − *x*, *y*, −*z*.

There are two half mol­ecules of each kind in the asymmetric unit of (I). Both Si_5_Cl_12_ mol­ecules are completed by crystallographic twofold symmetry, with the rotation axes orientated in the [110] and [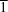
10] directions and the central Si atom located on the axis (Fig. 2 ▸). One of the benzene mol­ecules is located on a twofold rotation axis propagating along the *a* or *b* axes with two C—H groups located on this rotation axis. The second benzene mol­ecule has all atoms on general positions: it is disordered over two equally occupied orientations about a twofold rotation axis running in the [100] and [010] directions.

## Supra­molecular features   

**Figure 3:**
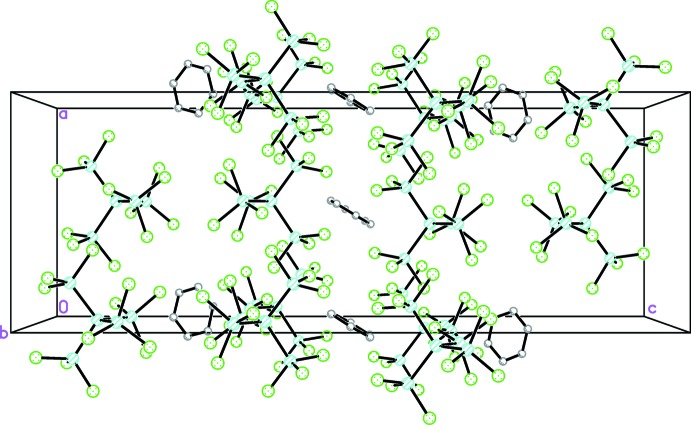
Packing diagram of the title compound viewed down [010].

A view of the mol­ecular packing of (I) (Fig. 3 ▸) reveals that the benzene mol­ecules fill the voids between the dodeca­chloro­penta­silane mol­ecules. There are no identified directional inter­molecular inter­actions.

## Database survey   

There are two already known structures of dodeca­chloro­penta­silane: first, there is pure Si_5_Cl_12_ (Meyer-Wegner *et al.*, 2011 ▸; CCDC deposition number 793308) and second, a co-crystal with silicon tetra­chloride (Fleming, 1972 ▸; CCDC deposition number 1592571). In each of these structures, the Si_5_Cl_12_ mol­ecule is located on a special position. As noted above, in (I), both mol­ecules in the asymmetric unit are found on a twofold rotation axis. Compound (II) also crystallizes with two mol­ecules in the asymmetric unit. One of them is located on a threefold rotation axis and the other is disordered about a special position of site symmetry 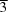. In the second mol­ecule, it is noteworthy that only the Si atoms carrying the Cl atoms are disordered: the central Si atom and the Cl atoms themselves are not disordered. In (III), the Si_5_Cl_12_ mol­ecule is located on a special position of site symmetry 23. The central Si atom is located at the inter­section of the twofold and the threefold rotation axes (the twofold rotation axis coincides with a 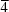 axis). The Si—Si and Si—Cl bond lengths in all three structures agree well (Table 1 ▸).

## Synthesis and crystallization   

The perchlorinated neo­penta­silane (I) was synthesized according to a literature procedure (Kaczmarczyk & Urry, 1960 ▸). Single crystals of Si(SiCl_3_)_4_·C_6_H_6_ were grown from a solution of Si(SiCl_3_)_4_ in benzene after one week at room temperature.

**Si(SiCl_3_)_4_** (**I**). ^29^Si{^1^H}NMR (C_6_D_6_, external TMS): δ = −80.9 [*Si*(SiCl_3_)_4_], δ = 3.5 [Si(*Si*Cl_3_)_4_].

## Refinement   

Crystal data, data collection and structure refinement details are summarized in Table 2 ▸. The H atoms were refined using a riding model with C—H = 0.95 Å and with *U*_iso_(H) = 1.2*U*_eq_(C). One of the benzene mol­ecules is disordered over two equally occupied orientations: its carbon atoms were isotropically refined. The C—C distances in the non-disordered benzene mol­ecule were restrained to 1.390 (2) Å. The crystal chosen for data collection was found to crystallize as a racemic twin.

## Supplementary Material

Crystal structure: contains datablock(s) I, global. DOI: 10.1107/S2056989020000900/hb7874sup1.cif

Structure factors: contains datablock(s) I. DOI: 10.1107/S2056989020000900/hb7874Isup2.hkl

CCDC reference: 1979631

Additional supporting information:  crystallographic information; 3D view; checkCIF report

## Figures and Tables

**Table 1:** Bond lengths (Å) in the different structures containing Si_5_Cl_12_ mol­ecules For (I), mean values of the two mol­ecules are given. For (II), mean values of the non-disordered mol­ecule are given. Because of the high symmetry of (III), there is only one value for each bond length.

	Si—Si	Si—Cl
(I)	2.324	2.019
(II)	2.340	2.026
(III)	2.332 (9)	1.994 (7)

**Table 2:** Experimental details

Crystal data	
Chemical formula	Cl_12_Si_5_·C_6_H_6_
*M*_r_	643.96
Crystal system, space group	Tetragonal, *P*4_1_22
Temperature (K)	173
*a*, *c* (Å)	11.9633 (4), 33.7848 (16)
*V* (Å^3^)	4835.3 (4)
*Z*	8
Radiation type	Mo *K*α
μ (mm^−1^)	1.62
Crystal size (mm)	0.28 × 0.18 × 0.16

Data collection	
Diffractometer	Stoe *IPDS* II two-circle
Absorption correction	Multi-scan (*X-AREA*; Stoe & Cie, 2001 ▸)
*T*_min_, *T*_max_	0.803, 1.0
No. of measured, independent and observed [*I* > 2σ(*I*)] reflections	39180, 5189, 4790
*R*_int_	0.047
(sin θ/λ)_max_ (Å^−1^)	0.642

Refinement	
*R*[*F* ^2^ > 2σ(*F* ^2^)], *wR*(*F* ^2^), *S*	0.027, 0.075, 1.08
No. of reflections	5189
No. of parameters	207
No. of restraints	5
H-atom treatment	H-atom parameters constrained
Δρ_max_, Δρ_min_ (e Å^−3^)	0.39, −0.42
Absolute structure	Flack *x* determined using 1905 quotients [(*I* ^+^)−(*I* ^−^)]/[(*I* ^+^)+(*I* ^−^)] (Parsons *et al.*, 2013 ▸)
Absolute structure parameter	0.48 (5)

Computer programs: *X-AREA* (Stoe & Cie, 2001 ▸), *SHELXS97* (Sheldrick, 2008 ▸), *XP* in *SHELXTL-Plus* (Sheldrick, 2008 ▸), *SHELXL2018* (Sheldrick, 2015 ▸) and *publCIF* (Westrip, 2010 ▸).

## supplementary crystallographic information

### Crystal data

**Table:**

Cl_12_Si_5_·C_6_H_6_	*D*_x_ = 1.769 Mg m^−^^3^
*M**_r_* = 643.96	Mo *K*α radiation, λ = 0.71073 Å
Tetragonal, *P*4_1_22	Cell parameters from 62511 reflections
*a* = 11.9633 (4) Å	θ = 1.9–27.6°
*c* = 33.7848 (16) Å	µ = 1.62 mm^−^^1^
*V* = 4835.3 (4) Å^3^	*T* = 173 K
*Z* = 8	Block, colourless
*F*(000) = 2528	0.28 × 0.18 × 0.16 mm

### Data collection

**Table:**

Stoe IPDS II two-circle diffractometer	4790 reflections with *I* > 2σ(*I*)
ω scans	*R*_int_ = 0.047
Absorption correction: multi-scan (X-AREA; Stoe & Cie, 2001)	θ_max_ = 27.1°, θ_min_ = 1.8°
*T*_min_ = 0.803, *T*_max_ = 1.0	*h* = −14→15
39180 measured reflections	*k* = −15→15
5189 independent reflections	*l* = −43→43

### Refinement

**Table:**

Refinement on *F*^2^	Hydrogen site location: mixed
Least-squares matrix: full	H-atom parameters constrained
*R*[*F*^2^ > 2σ(*F*^2^)] = 0.027	*w* = 1/[σ^2^(*F*_o_^2^) + (0.0375*P*)^2^ + 2.8317*P*] where *P* = (*F*_o_^2^ + 2*F*_c_^2^)/3
*wR*(*F*^2^) = 0.075	(Δ/σ)_max_ = 0.001
*S* = 1.08	Δρ_max_ = 0.39 e Å^−^^3^
5189 reflections	Δρ_min_ = −0.42 e Å^−^^3^
207 parameters	Absolute structure: Flack *x* determined using 1905 quotients [(*I*^+^)-(*I*^-^)]/[(*I*^+^)+(*I*^-^)] (Parsons *et al.*, 2013)
5 restraints	Absolute structure parameter: 0.48 (5)
Primary atom site location: structure-invariant direct methods	

### Special details

**Table:**

Geometry. All esds (except the esd in the dihedral angle between two l.s. planes) are estimated using the full covariance matrix. The cell esds are taken into account individually in the estimation of esds in distances, angles and torsion angles; correlations between esds in cell parameters are only used when they are defined by crystal symmetry. An approximate (isotropic) treatment of cell esds is used for estimating esds involving l.s. planes.

### Fractional atomic coordinates and isotropic or equivalent isotropic displacement parameters (Å^2^)

**Table:**

	*x*	*y*	*z*	*U*_iso_*/*U*_eq_	Occ. (<1)
Si1	0.54973 (7)	0.45027 (7)	0.125000	0.0222 (3)	
Si2	0.38834 (7)	0.45056 (7)	0.08678 (2)	0.02447 (19)	
Si3	0.54972 (8)	0.29277 (8)	0.16530 (3)	0.0264 (2)	
Cl21	0.40377 (8)	0.34525 (8)	0.04060 (2)	0.0400 (2)	
Cl22	0.25695 (7)	0.39988 (8)	0.11990 (3)	0.0387 (2)	
Cl23	0.35776 (8)	0.60671 (7)	0.06658 (3)	0.0384 (2)	
Cl31	0.49743 (8)	0.15929 (7)	0.13366 (3)	0.0408 (2)	
Cl32	0.70602 (8)	0.26452 (9)	0.18579 (3)	0.0453 (2)	
Cl33	0.44514 (8)	0.31660 (9)	0.21128 (3)	0.0412 (2)	
Si4	0.04988 (7)	−0.04988 (7)	0.125000	0.0225 (3)	
Si5	0.05165 (7)	0.11311 (7)	0.16232 (2)	0.02420 (19)	
Si6	0.20502 (8)	−0.05209 (8)	0.08356 (3)	0.0263 (2)	
Cl51	−0.05058 (7)	0.09833 (8)	0.20938 (2)	0.0382 (2)	
Cl52	−0.00287 (8)	0.24225 (7)	0.12875 (3)	0.0367 (2)	
Cl53	0.20867 (7)	0.14553 (8)	0.18124 (3)	0.0379 (2)	
Cl61	0.23134 (9)	0.10282 (8)	0.06166 (3)	0.0449 (2)	
Cl62	0.17856 (9)	−0.15973 (8)	0.03862 (2)	0.0414 (2)	
Cl63	0.34091 (8)	−0.10198 (9)	0.11430 (3)	0.0424 (2)	
C1	0.000000	0.3434 (6)	0.000000	0.100 (4)	
H1	0.000000	0.263981	0.000000	0.120*	
C2	0.0511 (5)	0.4024 (4)	0.03030 (13)	0.094 (2)	
H2	0.087169	0.363505	0.051231	0.113*	
C3	0.0495 (5)	0.5176 (4)	0.03001 (14)	0.090 (2)	
H3	0.083717	0.556596	0.051284	0.107*	
C4	0.000000	0.5786 (7)	0.000000	0.087 (3)	
H4	0.000000	0.657984	0.000000	0.104*	
C5	0.5409 (8)	0.8628 (10)	0.0236 (3)	0.051 (2)*	0.5
H5	0.570943	0.806236	0.040196	0.062*	0.5
C6	0.5561 (8)	0.9738 (11)	0.0335 (3)	0.050 (2)*	0.5
H6	0.594385	0.992910	0.057205	0.059*	0.5
C7	0.5158 (9)	1.0566 (8)	0.0092 (3)	0.054 (3)*	0.5
H7	0.527035	1.132987	0.015732	0.065*	0.5
C5'	0.5145 (10)	0.8339 (8)	0.0086 (3)	0.057 (3)*	0.5
H5'	0.526088	0.757264	0.014687	0.068*	0.5
C6'	0.5559 (9)	0.9177 (12)	0.0335 (3)	0.058 (2)*	0.5
H6'	0.594991	0.897299	0.056942	0.069*	0.5
C7'	0.5410 (10)	1.0290 (11)	0.0247 (4)	0.062 (3)*	0.5
H7'	0.569534	1.085379	0.041783	0.074*	0.5

### Atomic displacement parameters (Å^2^)

**Table:**

	*U*^11^	*U*^22^	*U*^33^	*U*^12^	*U*^13^	*U*^23^
Si1	0.0213 (4)	0.0213 (4)	0.0241 (6)	0.0004 (5)	0.0008 (3)	0.0008 (3)
Si2	0.0245 (4)	0.0238 (4)	0.0251 (4)	0.0000 (3)	−0.0010 (3)	0.0009 (3)
Si3	0.0252 (4)	0.0247 (4)	0.0294 (4)	0.0001 (4)	−0.0007 (3)	0.0046 (3)
Cl21	0.0521 (5)	0.0364 (5)	0.0316 (4)	−0.0014 (4)	0.0003 (4)	−0.0084 (3)
Cl22	0.0269 (4)	0.0476 (5)	0.0418 (4)	−0.0054 (4)	0.0051 (3)	0.0042 (4)
Cl23	0.0464 (5)	0.0278 (4)	0.0411 (4)	0.0058 (4)	−0.0079 (4)	0.0060 (3)
Cl31	0.0452 (5)	0.0268 (4)	0.0503 (5)	−0.0048 (4)	−0.0003 (4)	−0.0032 (4)
Cl32	0.0317 (4)	0.0461 (5)	0.0579 (5)	0.0068 (4)	−0.0113 (4)	0.0116 (4)
Cl33	0.0406 (5)	0.0529 (6)	0.0302 (4)	−0.0059 (4)	0.0065 (4)	0.0031 (4)
Si4	0.0214 (4)	0.0214 (4)	0.0245 (6)	0.0000 (5)	0.0006 (3)	0.0006 (3)
Si5	0.0236 (4)	0.0242 (4)	0.0248 (4)	−0.0008 (3)	−0.0010 (3)	−0.0011 (3)
Si6	0.0248 (4)	0.0259 (4)	0.0282 (4)	0.0001 (4)	0.0038 (3)	0.0010 (3)
Cl51	0.0366 (5)	0.0486 (5)	0.0295 (4)	0.0010 (4)	0.0072 (3)	−0.0013 (4)
Cl52	0.0436 (5)	0.0268 (4)	0.0398 (4)	0.0039 (4)	−0.0050 (4)	0.0042 (3)
Cl53	0.0282 (4)	0.0428 (5)	0.0429 (4)	−0.0063 (4)	−0.0069 (3)	−0.0033 (4)
Cl61	0.0489 (5)	0.0325 (4)	0.0533 (5)	−0.0063 (4)	0.0126 (4)	0.0108 (4)
Cl62	0.0520 (5)	0.0404 (5)	0.0317 (4)	0.0053 (4)	0.0022 (4)	−0.0084 (4)
Cl63	0.0274 (4)	0.0505 (5)	0.0494 (5)	0.0067 (4)	−0.0039 (4)	0.0003 (4)
C1	0.074 (6)	0.043 (4)	0.184 (11)	0.000	0.056 (7)	0.000
C2	0.068 (4)	0.147 (7)	0.067 (4)	0.011 (4)	0.001 (3)	0.055 (4)
C3	0.070 (4)	0.138 (6)	0.060 (3)	−0.026 (4)	0.006 (3)	−0.037 (4)
C4	0.067 (5)	0.068 (5)	0.125 (8)	0.000	0.040 (5)	0.000

### Geometric parameters (Å, º)

**Table:**

Si1—Si2	2.3227 (11)	Si6—Cl61	2.0202 (13)
Si1—Si2^i^	2.3228 (11)	C1—C2	1.3855 (18)
Si1—Si3^i^	2.3246 (11)	C1—C2^iii^	1.3855 (18)
Si1—Si3	2.3247 (11)	C1—H1	0.9500
Si2—Cl21	2.0138 (12)	C2—C3	1.378 (3)
Si2—Cl23	2.0221 (12)	C2—H2	0.9500
Si2—Cl22	2.0225 (12)	C3—C4	1.3824 (18)
Si3—Cl33	2.0150 (13)	C3—H3	0.9500
Si3—Cl31	2.0209 (13)	C4—H4	0.9500
Si3—Cl32	2.0223 (13)	C5—C6	1.382 (15)
Si4—Si5	2.3221 (11)	C5—H5	0.9500
Si4—Si5^ii^	2.3222 (11)	C6—C7	1.375 (14)
Si4—Si6	2.3250 (11)	C6—H6	0.9500
Si4—Si6^ii^	2.3251 (11)	C7—H7	0.9500
Si5—Cl51	2.0138 (12)	C5'—C6'	1.399 (15)
Si5—Cl53	2.0218 (12)	C5'—H5'	0.9501
Si5—Cl52	2.0243 (12)	C6'—C7'	1.376 (15)
Si6—Cl62	2.0158 (13)	C6'—H6'	0.9500
Si6—Cl63	2.0194 (13)	C7'—H7'	0.9500
			
Si2—Si1—Si2^i^	107.85 (6)	C2—C1—C2^iii^	118.8 (7)
Si2—Si1—Si3^i^	110.38 (3)	C2—C1—H1	120.6
Si2^i^—Si1—Si3^i^	109.07 (3)	C2^iii^—C1—H1	120.6
Si2—Si1—Si3	109.07 (3)	C3—C2—C1	119.9 (6)
Si2^i^—Si1—Si3	110.37 (3)	C3—C2—H2	120.1
Si3^i^—Si1—Si3	110.06 (7)	C1—C2—H2	120.1
Cl21—Si2—Cl23	109.46 (5)	C2—C3—C4	122.6 (6)
Cl21—Si2—Cl22	108.20 (6)	C2—C3—H3	118.7
Cl23—Si2—Cl22	108.85 (6)	C4—C3—H3	118.7
Cl21—Si2—Si1	110.71 (5)	C3^iii^—C4—C3	116.3 (7)
Cl23—Si2—Si1	109.84 (5)	C3^iii^—C4—H4	121.9
Cl22—Si2—Si1	109.75 (4)	C3—C4—H4	121.9
Cl33—Si3—Cl31	109.11 (6)	C6—C5—C5^iv^	106.0 (6)
Cl33—Si3—Cl32	109.50 (6)	C6—C5—H5	119.4
Cl31—Si3—Cl32	109.58 (6)	C5^iv^—C5—H5	134.6
Cl33—Si3—Si1	109.69 (5)	C5—C6—C7	120.0 (9)
Cl31—Si3—Si1	109.31 (5)	C5—C6—C7^iv^	104.5 (8)
Cl32—Si3—Si1	109.63 (5)	C5—C6—H6	120.0
Si5—Si4—Si5^ii^	108.07 (6)	C7—C6—H6	120.0
Si5—Si4—Si6	109.22 (3)	C7^iv^—C6—H6	135.5
Si5^ii^—Si4—Si6	110.07 (3)	C7^iv^—C7—C6	133.9 (6)
Si5—Si4—Si6^ii^	110.08 (3)	C6—C7—C6^iv^	103.4 (9)
Si5^ii^—Si4—Si6^ii^	109.22 (3)	C7^iv^—C7—H7	105.9
Si6—Si4—Si6^ii^	110.15 (7)	C6—C7—H7	120.2
Cl51—Si5—Cl53	109.35 (5)	C6^iv^—C7—H7	136.4
Cl51—Si5—Cl52	108.28 (6)	C5'^iv^—C5'—C6'	134.3 (6)
Cl53—Si5—Cl52	109.28 (6)	C5'^iv^—C5'—H5'	105.1
Cl51—Si5—Si4	110.45 (5)	C6'—C5'—H5'	120.6
Cl53—Si5—Si4	109.95 (5)	C6'^iv^—C5'—H5'	136.3
Cl52—Si5—Si4	109.50 (5)	C7'—C6'—C5'	121.1 (10)
Cl62—Si6—Cl63	108.97 (6)	C7'—C6'—H6'	119.4
Cl62—Si6—Cl61	109.56 (6)	C5'—C6'—H6'	119.4
Cl63—Si6—Cl61	109.51 (6)	C5'^iv^—C6'—H6'	134.0
Cl62—Si6—Si4	109.60 (5)	C6'—C7'—C7'^iv^	104.6 (7)
Cl63—Si6—Si4	109.65 (5)	C6'—C7'—H7'	120.6
Cl61—Si6—Si4	109.54 (5)	C7'^iv^—C7'—H7'	134.8
			
C2^iii^—C1—C2—C3	0.6 (4)	C7^iv^—C6—C7—C6^iv^	−1 (2)
C1—C2—C3—C4	−1.2 (9)	C5'^iv^—C5'—C6'—C7'	0 (4)
C2—C3—C4—C3^iii^	0.6 (4)	C6'^iv^—C5'—C6'—C7'	0.0 (10)
C5^iv^—C5—C6—C7	−1.7 (13)	C6'^iv^—C5'—C6'—C5'^iv^	0 (3)
C5^iv^—C5—C6—C7^iv^	−1.2 (10)	C5'—C6'—C7'—C7'^iv^	−0.1 (14)
C5—C6—C7—C7^iv^	2 (3)	C5'^iv^—C6'—C7'—C7'^iv^	−0.2 (11)
C5—C6—C7—C6^iv^	1.0 (9)		

Symmetry codes: (i) −*y*+1, −*x*+1, −*z*+1/4; (ii) −*y*, −*x*, −*z*+1/4; (iii) −*x*, *y*, −*z*; (iv) −*x*+1, *y*, −*z*.
